# Personal Hygiene and Methicillin-resistant *Staphylococcus aureus* Infection

**DOI:** 10.3201/eid1203.050625

**Published:** 2006-03

**Authors:** George Turabelidze, Mei Lin, Barbara Wolkoff, Douglas Dodson, Stephen Gladbach, Bao-Ping Zhu

**Affiliations:** * Missouri Department of Health and Senior Services, Jefferson City, Missouri, USA

**Keywords:** hygiene, MRSA, outbreak, research

## Abstract

Methicillin-resistant *Staphylococcus aureus* (MRSA) infections outside the healthcare setting are an increasing concern. We conducted a case-control study to investigate an MRSA outbreak during 2002–2003 in a Missouri prison and focused on hygiene factors. Information on sociodemographic characteristics, medical history, and hygiene practices of study participants was collected by interview and medical record review. Logistic regression was used to evaluate MRSA infection in relation to hygiene factors individually and as a composite hygiene score; potential confounding factors were controlled. Selected MRSA isolates were analyzed by pulsed-field gel electrophoresis (PFGE). MRSA infection was significantly associated with a low composite hygiene score. Transmission among prison inmates appeared to be responsible for this outbreak. PFGE analysis showed that isolates were indistinguishable and associated with community-onset MRSA infections in other US prisons. Improving hygiene practices and environmental conditions may help prevent and interrupt future MRSA outbreaks in prison settings.

## Abstract 2

Improving personal hygienic practices may prevent and control MRSA outbreaks.

In recent years, outbreaks of methicillin-resistant *Staphylococcus aureus* (MRSA) infection have been reported in different settings, including among athletic teams and military recruits, as well as in nursing homes and correctional facilities (*1*–*5*). A number of risk factors for MRSA infection have been identified in these studies, including antimicrobial drug use, close contact with persons colonized with MRSA, and barriers to medical care. Although some outbreak investigations have pointed to personal hygiene as a risk factor for MRSA infection (*1*,*4*), few studies identified specific personal hygiene practices associated with increased risk.

In June 2003, an outbreak of MRSA infection at a women's correctional facility (prison X) was reported to the Missouri Department of Health and Senior Services. As part of the investigation and control of this outbreak, a case-control study of risk factors for MRSA infection was conducted, with a focus on personal hygiene factors. In addition, a laboratory investigation of the specimens collected from the infected persons was conducted to identify the strain of *S. aureus* implicated in the outbreak by pulsed-field gel electrophoresis (PFGE). This report summarizes the results of the investigation.

## Materials and Methods

### Case-Control Study

A case-control study was conducted to examine risk factors for MRSA infection in this outbreak, with a focus on personal hygiene factors. A case was defined as an inmate at prison X with culture-confirmed MRSA infection of skin or soft tissue diagnosed between January 1, 2002, and May 30, 2003. Controls were randomly chosen at the same prison with a systematic random sampling scheme from inmates who never experienced illness compatible with MRSA infection during the study period, and whose physical examination at the time of the investigation showed no evidence of MRSA infection. Case-patients and controls who reported having skin or soft tissue infection at prison admission were excluded. A trained interviewer administered a face-to-face interview with a standard questionnaire, and a physician performed a brief physical examination at the time of the interview. The interviewer collected information about sociodemographic characteristics, relevant medical history, personal hygiene factors (including hand washing, shower, laundry practices, and sharing personal items), use of gymnasium and barbershop, and attending educational classes. Medical records for the case-patients were obtained from the prison clinic where they sought care. Additionally, information regarding the history of antimicrobial drug use, chronic medical conditions, history of hospitalization, and history of skin infection at prison arrival was obtained through the interview and medical record review. Being overweight in this study was defined as a body mass index (BMI, weight in kilograms divided by height in meters squared) >25 (*6*).

To evaluate an overall effect of personal hygiene practice on MRSA infection, a composite hygiene score was created on the basis of the sum of scores of 3 individual hygiene practices, including frequency of hand washing per day (1 = <6 times, 2 = 6–12 times, 3 = >12 times), frequency of a shower per week (1 = <7 times, 2 = 7–13 times, 3 = >13 times), and number of personal items shared with other inmates (1 = >2 items, 2 = 1 item, 3 = none). In this manner, lower composite hygiene scores indicated poorer personal hygiene practices. For this study, a composite hygiene score <6, which corresponds to the 25th percentile for the distribution of the composite score, was categorized as poor personal hygiene practice. Sociodemographic characteristics, medical conditions, hygiene practices, and other potential risk factors were compared between patients and controls by using the χ^2^ test or Fisher's exact test (*7*). Logistic regression was used to evaluate crude and adjusted odds ratios (aORs) and their associated 95% confidence intervals (CIs) for MRSA infection in relation to individual hygiene factors separately; we controlled for patient age, race, educational level, being overweight, and skin condition before arrival at the prison. The final logistic regression model included the composite hygiene score and the covariates listed above. Model parameters were estimated by using the maximum likelihood method; their statistical significance was assessed by using the Wald statistic (*8*). All statistical analyses were performed with SAS version 9.1 (SAS, Cary, NC, USA) (*9*).

### Laboratory Investigation

Genotyping on selected MRSA isolates was performed by using PFGE with *SmaI*-digested DNA. Gels were analyzed with BioNumerics software (Applied Maths, Kortrijik, Belgium) as described by McDougal et al. (*10*). Pulsed-field types were defined in a national database as having >80% similarity in a dendrogram derived from the unweighted pair group method with arithmetic averages and based on Dice coefficients. Band position tolerance and optimization were set at 1.25% and 0.5%, respectively.

Written consent was obtained from all study participants. The Institutional Review Board at the Missouri Department of Health and Senior Services reviewed and approved this study.

## Results

**Figure 1:**
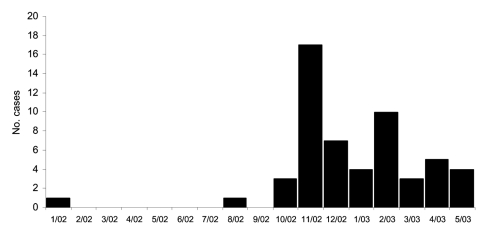
Onset dates of culture-confirmed cases of methicillin-resistant *Staphylococcus aureus*, prison X, Missouri, 2002–2003.

A total of 55 culture-confirmed MRSA cases occurred at prison X during the study period (Figure 1). Of those case-patients, 30 (55%) were available for interview at the time of the investigation; 82 inmates of the same prison who had no MRSA infection were randomly selected as controls. All 30 patients participating in the study had skin or wound infections. Two controls were excluded from the study; 1 had folliculitis and the other provided insufficient data during the interview. The final dataset contained 30 cases and 80 controls.

The average length of stay in the prison, calculated from admission to the time of outbreak investigation, was significantly lower for case-patients (22.8 months) than for controls (38.9 months). The mean time from prison admission to culture confirmation of MRSA infection was 624 days (range 48–2,303), and the median was 415 days; 27 (90%) of 30 had culture confirmation >90 days after prison admission.

Compared with controls, patients were younger (mean age 34.5 vs. 41.5 years), more likely to be African American or American Indian, and less likely to have attended college (Table 1). No statistically significant differences between cases and controls occurred with regard to chronic medical conditions, hospitalization during the 6 months before prison admission, and intravenous drug use. Case-patients were more likely than controls to report abnormal skin conditions (i.e., infection, dermatitis, eczema; 13.3% vs. 3.8%) in their medical history, but the difference was marginally significant (p = 0.09). Patients and controls did not differ in antimicrobial drug use (topical or systemic) in the 3 months before prison admission. Patients were significantly more likely to be overweight (56.7% vs. 23.8%, p<0.01) than controls (Table 1). Patients and controls did not differ significantly in use of the gymnasium and barbershop and in attending classes.

**Table 1:** Characteristics of patients with methicillin-resistant *Staphylococcus aureus* infection and controls, prison X, Missouri, 2002–2003*

Characteristics	Case-patients, % (n = 30)	Controls, % (n = 80)	Crude OR (95% CI)
Age, y			
20–34	60	20	6.00 (1.47–24.45)
35–49	30	60	1.00 (0.24–4.15)
>50	10	20	1.00
Race			
Caucasian	20	51.3	1.00
Non-Caucasian	80	48.8	4.21 (1.55–11.39)
Educational level			
No college†	86.7	72.5	2.47 (0.77–7.88)
College	13.3	27.5	1.00
Overweight‡			
Yes	56.7	23.8	4.20 (1.73–10.19)
No	43.3	76.3	1.00
Antimicrobial drug use in the 3 months before imprisonment			
Yes	13.3	7.5	1.90 (0.50–7.26)
No	86.7	92.5	1.00
Abnormal skin condition before arriving			
Yes	13.3	3.8	3.95 (0.83–18.82)
No	86.7	96.3	1.00

*OR, odds ratio; CI, confidence interval. Other characteristics examined included chronic medical condition, daily medication use for treating chronic conditions, hospitalization 6 months before prison admission, intravenous drug use, use of gymnasium or barbershop, and attending educational classes. These characteristics did not differ significantly between patients and controls. †Included no high school, graduation from high school, or general educational development. ‡Defined as a body mass index >25.

When personal hygiene factors were examined for cases and controls (Table 2), patients were more likely than controls to share personal products (e.g., cosmetic items, lotion, bedding, toothpaste, headphones), especially nail clippers (26.7% vs. 10%, p = 0.04) and shampoo (13.3% vs. 1.3%, p = 0.02), with other inmates. Patients were also less likely than controls to wash personal items (80.0% vs. 88.8%, p<0.01) or bed linens (26.7% vs. 52.5%, p<0.01) themselves instead of using the prison laundry. Additionally, patients tended to wash their hands and take showers less often.

**Table 2:** Distribution of hygiene factors among persons with methicillin-resistant *Staphylococcus aureus* and controls, prison X, Missouri, 2002–2003*

Characteristics	Case-patients, % (n = 30)	Controls, % (n = 80)	Adjusted OR (95% CI)
Always wash personal items themselves			
Yes	80	88.8	1.00
No	20	1.3	23.89 (2.07–275.88)
Not sure	0	10	
Always wash bed linen themselves			
Yes	26.7	52.5	1.00
No	73.3	37.5	3.88 (1.25–12.01)
Not sure	0	10	
Share any product (cosmetics, nail clipper, shaver, bedding, etc.)			
Yes	60	37.5	1.79 (0.64–4.99)
No	40	62.5	1.00
No. shared products			
>2	33.3	17.5	2.15 (0.63–7.39)
1	26.7	20	1.49 (0.44–5.11)
0	40	62.5	1.00
Share shampoo			
Yes	13.3	1.3	3.32 (0.30–36.67)
No	86.7	98.8	1.00
Share nail clipper			
Yes	26.7	10	3.03 (0.85–10.74)
No	73.3	90	1.00
Wash hands, times per day			
<6	6.7	2.5	2.17 (0.15–31.93)
6–12	50	32.5	3.27 (1.10– 9.76)
>12	43.3	65	1.00
Showers per week			
<7	10	5	5.01 (0.53–47.11)
7–13	66.7	53.8	2.68 (0.85–8.46)
>14	23.3	41.3	1.00

*OR, odds ratio; CI, confidence interval. Adjusted ORs were from separate logistic regression models in which the individual hygiene factor and age, race, educational level, overweight (body mass index >25), and abnormal skin conditions before arrival were included.

When personal hygiene factors were examined individually by logistic regression while controlling for sociodemographic and other risk factors, inmates who used the prison laundry to wash their personal items (aOR 23.89, 95% CI 2.1–275.9) or bed linens (aOR 3.9, 95% CI 1.3–12.0) were more likely to have an MRSA infection than inmates who washed those items themselves. Because 8 controls were unsure about this question and were excluded from this analysis, we performed a sensitivity analysis by assigning them to the group using the prison laundry to produce the most conservative estimates. In so doing, washing bed linen in the prison laundry still showed a marginally significant association with MRSA infection (aOR 2.84, 95% CI 0.96–8.42), whereas the effect of washing personal items in the prison laundry was largely diminished (aOR 1.74, 95% CI 0.46–6.60).

The risk for MRSA infection also increased with lower frequency of hand washing per day and showers per week. Inmates who washed their hands 6–12 times (aOR 3.27, 95% CI 1.10–9.76) and <6 times (aOR 2.17, 95% CI 0.15–31.93) per day had an increased risk for infection compared with that of inmates who washed their hands >12 times per day. Inmates who took <7 showers per week (aOR 5.01, 95% CI 0.53–47.11) and those who took 7–13 showers per week (aOR 2.68, 95% CI 0.85–8.46) had an increased risk for infection compared with that of inmates who took >14 showers per week. Inmates who shared personal products with other inmates tended to have an increased risk for MRSA infection compared with inmates who did not share their personal products with others.

When the data were examined by using the composite hygiene score, a significantly higher proportion of case-patients than controls had lower hygiene scores (<6) (46.7% vs. 20.0%, p<0.01). When the relationship between MRSA infection and the composite hygiene score was evaluated while simultaneously controlling for sociodemographic characteristics and other risk factors (Table 3), inmates who had poor composite hygiene scores (<6) had a significantly higher risk for MRSA infection compared with those who had higher composite hygiene scores (aOR 3.14, 95% CI 1.1–8.93). The risk for infection also tended to increase with being younger, overweight, and nonwhite, and having a history of an abnormal skin condition. When case-patients were asked how they cared for their skin infections, 53.3% reportedly did not always cover their skin lesions, 36.7% did not have extra dressing for changing when needed, 56.7% picked their sores, and 36.7% did not change their dressings daily.

**Table 3:** Adjusted odds ratios (ORs) and 95% confidence intervals (CIs) associated with risk factors for infection with methicillin-resistant *Staphylococcus aureus*, prison X, Missouri, 2002–2003*

Characteristics	Adjusted OR (95% CI)
Composite hygiene score	
<6	3.14(1.10–8.93)
>6	1.00
Age, y	
20–34	3.57 (0.70–18.19)
35–49	0.75 (0.16–3.60)
>50	1.00
Race	
Caucasian	1.00
Non-Caucasian	2.21 (0.70–6.96)
Educational level	
No college†	1.22 (0.30–4.92)
College	1.00
Overweight‡	
Yes	2.48 (0.86–7.14)
No	1.00
Abnormal skin condition before arriving	
Yes	2.65 (0.47–15.07)
No	1.00

*Adjusted ORs were calculated from a logistic regression model that simultaneously included all of the risk factors shown. †Included no high school, graduation from high school, or general educational development. ‡Defined as a body mass index >25.

**Figure 2:**
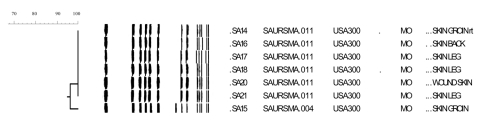
Dendrogram of the general relatedness (scale bar) of a sample of methicillin-resistant *Staphylococcus aureus* isolates based on pulsed-field gel electrophoresis of *Sma*I-digested DNA and comparisons of banding patterns using Dice similarity coefficients.

Analysis of PFGE results showed that 6 isolates had indistinguishable banding patterns and were identified as USA300.0114 (Figure 2). The banding pattern of a seventh isolate (SA15) differed by 1 band from the outbreak pattern.

## Discussion

In this case-control study of an MRSA outbreak in a prison setting, poor personal hygiene practices were significantly associated with an increased risk for MRSA infection after controlling for sociodemographic and other risk factors. This outbreak was likely caused by transmission inside the prison because 90% of the case-patients had culture confirmation at least 90 days after prison admission, and subtyping by PFGE showed that 6 of the 7 isolates tested had identical PFGE patterns and 1 differed by only 1 band. These isolates belonged to pulsed-field type USA300 lineage, which is associated with community-onset MRSA infections in other correctional facilities and community outbreaks (*11*).

Based on literature review, outbreaks of MRSA infection are thought to be caused by the complex interaction of the environment contaminated by MRSA, indiscriminate use of antimicrobial drugs, and personal hygiene factors (*12*,*13*). In a crowded, institutionalized setting such as a prison, the interplay of such factors is more pronounced. As a result, many outbreaks have occurred in such settings (*1*,*14*). Hospital environmental surfaces, healthcare worker gowns, and patient-care items contaminated by patients infected or colonized with MRSA have been shown to pose significant risks for MRSA transmission (*12*,*15*). Boyce et al. (*16*) found that 73% of hospital rooms containing patients infected with MRSA and 69% of rooms containing patients colonized with MRSA had environmental contamination. Research also showed that the nurses' gloves became contaminated 42% of the time after they touched surfaces contaminated with the bacteria. Potential transmission of MRSA infection through contaminated surfaces and shared items was identified in a rural community by Baggett et al. (*17*). In a community-based study, Calfee et al. (*18*) demonstrated that close contact with a person colonized or infected with MRSA resulted in a 7.5-fold greater risk of becoming colonized with MRSA. Persons colonized with MRSA also have an increased risk for MRSA infection (*19*,*20*). Based on the results of these studies and observations in this study, one can conclude that a prison environment can be easily contaminated by MRSA. Improved personal hygiene may provide protection for inmates living and working in such contaminated environments.

In this outbreak, a complex set of factors likely contributed to the spread of infection. These factors include improper care of infected skin lesions by inmates, poor personal hygiene by inmates, and close contact in confined space.

Risk factors in an MRSA outbreak in a Georgia prison were previous antimicrobial drug use, self-draining of boils, skin lacerations, washing clothes by hand, sharing soap, and recent arrival at the prison; risk factors in an MRSA outbreak in a Texas prison included previous skin infections and recent contact with MRSA-infected persons (*1*). Nguyen et al. (*21*) found that sharing soap was associated with recurrent MRSA infections in a football team. Our finding that sharing personal hygiene items is a risk factor for MRSA infection is consistent with these observations. The use of antimicrobial drugs within 3 months before incarceration did not appear to be a significant risk factor in our investigation, and prior skin conditions, including infections, were only marginally associated with MRSA infection by univariate analysis.

Previous research indicated that patients with community-acquired MRSA infections are usually children and young adults (*13*). Our study also indicates that younger age appears to be associated with an increased risk for MRSA infection in a prison setting. The increased risk associated with younger inmates in our study was likely due to a more active lifestyle, which predisposes them to skin abrasions. These abrasions serve as the ports of entry for bacterial infection.

Our study found that being overweight was a risk factor for MRSA infection. This finding was consistent with the results of a study by Kazakova et al. (*11*), who reported a significantly higher risk for MRSA infection in football players with a higher BMI. In a study on postoperative mediastinitis caused by methicillin-susceptible *S. aureus* conducted by Duke University Medical Center, the only independent risk factor was obesity (*22*). Persons who are overweight may have different patterns of skin colonization with MRSA, which puts them at greater risk for MRSA infection.

Previous studies indicated that certain racial and ethnic minority groups may have higher rates of colonization and infection with community-acquired MRSA (*23*,*24*). In our study, being nonwhite (African American and American Indian) was a significant risk factor for acquiring MRSA infection before controlling for other risk factors. However, after controlling for other risk factors, this association was no longer significant.

Several limitations should be considered when interpreting the findings of this study. First, the MRSA cases were diagnosed between January 1, 2002, and May 30, 2003. However, all questionnaires were administered in May 2003. The length of time from symptom onset to the date of interview was 0.5–17 months (median 4.9). Therefore, there could be recall bias about risk factors, especially for inmates who were interviewed long after symptom onset. Second, all study participants were incarcerated adult women. Therefore, the study findings may not be applicable to other populations. Third, personal hygiene factors emerged as a leading factor for MRSA transmission in a prison setting. Whether this finding can be applied to other settings (e.g., hospitals, nursing homes, and communities) needs further investigation.

The findings of this study underscore the importance of the targeted education efforts to control MRSA outbreaks. Education about MRSA infection, especially the importance of proper personal hygiene, should be an integral part of efforts to eliminate and prevent MRSA infections and outbreaks. Such measures may be important in reducing the spread of MRSA in prison settings, where inherent rules and regulations complicate the implementation of certain control measures.
